# Operator Experience Does Not Affect the Reliability of Universal Adhesives’ Bond Strength; Adhesive Type Does

**DOI:** 10.3290/j.jad.c_2675

**Published:** 2026-06-23

**Authors:** Marwa Bawazir, Sibel A. Antonson

**Affiliations:** a Marwa Bawazir Assistant Professor, Department of Restorative Dentistry, Faculty of Dentistry, King Abdulaziz University, Jeddah, Saudi Arabia. Hypothesis development, experimental design, performed the experiments in partial fulfillment of requirements for MSc degree, wrote the manuscript, and proofread the manuscript.; b Sibel A. Antonson Professor, Department of Prosthodontics, Biruni University, Faculty of Dentistry, Istanbul, Turkey. Idea, hypothesis development, experimental design, proofreading the manuscript, consulted on statistical evaluation, and contributed substantially to the manuscript.

**Keywords:** adhesive system, dentin, dentin surface condition, operator variability, reliability, SEM analysis, shear bond strength

## Abstract

**Purpose:**

To determine the influence of operators’ experience, dentin moisture condition (DMC), and adhesive type on shear bond strength, and evaluate the consistency of adhesive performance under these conditions to demonstrate clinical reliability.

**Methods and Materials:**

Six hundred sound dentin surfaces were randomly divided by five dentists with at least 10 years of experience (n = 300/5 dentists) and five first-year dental students without dental experience (n = 300/5 students). Each operator applied universal (UA) (Adhese Universal, Ivoclar) and etch-and-rinse (ERA) (OptiBond FL, Kerr) adhesives (n = 60/adhesive type) before and after receiving demonstration on the manufacturers’ recommended application techniques by the principal investigator (PI), on wet, moist, and dry dentin surfaces (n = 5/DMC). DMCs were obtained by the PI before the operators applied the adhesives. The PI applied composite restorations (Tetric EvoCeram Bulk Fill, Ivoclar) and polymerized for 20 s. Shear bond strength (SBS) test was conducted after 24 h storage at 37°C, in 100% humidity. Boxplot, Log Worth, and Tukey HSD were used for statistics.

**Results:**

The median SBS of ERA obtained by dentists was 17.79 MPa with an interquartile range (IQR) of 19.61, while students showed a lower median of 13.52 MPa with IQR of 15.81. The UA showed consistency in SBS and reduced variability across operators with median SBS by dentists and students (25.53 MPa and 24.96 MPa, respectively), with narrower IQRs of 10.6 and 7.3, respectively. SBS of UA was significantly higher than ERA (*P *< 0.001). UA obtained 7.38 MPa ± 0.89 greater SBS than ERA. While no difference in SBS by operator was observed for UA, dentists obtained 1.65 MPa ± 0.73 greater SBS than students with ERA. Dry and moist dentin surface conditions yielded higher SBS values compared to wet surfaces (*P* < 0.001 and *P* < 0.002, respectively), with no significant differences observed between moist and dry surfaces.

**Conclusion:**

The tested UA provided reliable outcomes regardless of operator and DMC.

Composite resin restorations show excellent mechanical and optical properties that qualify them to mimic natural tooth structure and successfully restore missing dental hard tissues.^15,47^ However, these composite resin restorations rely on adhesives to efficiently bond to dentin and enamel, dentin being the variable substructure.^3^ Enamel is a highly mineralized tissue composed of 90% minerals, providing a consistent substructure for adhesive bonding. Conversely, dentin is a less mineralized tissue that comprises 50% minerals along with a large amount of organic material and water.^31,34^ Furthermore, besides the high organic content, dentin has distinctive biological characteristics, including the presence of dentinal fluid inside the dentinal tubules, the permeability of dentin, and the formation of the smear layer.^4,19,25^ This structure inherently results in an inconsistent and less predictable substructure for bonding.^29^ The reliability of dental adhesives’ bond strength to dentin has been a consistent challenge throughout the evolution of dentin adhesive systems. Reliability in this context is defined as the consistency, dependability, and sustainability of the bond strength outcomes regardless of the clinical variables. Evaluating the reliability of the adhesive system gives clinicians confidence in their daily practices that eventually translates into assurance of adequate patient care, providing consistently solid restorations and increased productivity regardless of the clinical challenges encountered.

Dental adhesives have advanced considerably over the years. From the conventional three-step etch-and-rinse adhesive systems, which require multiple steps of acid etching, primer application, and resin-based adhesives, to the modern single-step universal adhesives, all these systems share a common goal of achieving optimal bond strength between composite restorations and the tooth structures.^28^ Etch-and-rinse adhesive systems necessitate the application of phosphoric acid to remove the smear layer, create micro-porosities, and expose dentin collagen fibers and enamel prisms. The primer and resin-based adhesive penetrate the conditioned enamel and dentin surfaces and integrate the hydrophobic composite resin into the hydrophilic tooth structure.^19,40^ For years, the three-step etch-and-rinse adhesive system was considered the gold standard for bonding composite resin restorations to enamel and dentin substrates.^18,21,24^ However, the multiple clinical bonding steps of etch-and-rinse adhesives introduced increased variability in the application technique and created susceptibility to operator error.^11^

The multi-step adhesive systems are also sensitive to different dentin moisture conditions. The exposed dentin collagen fibers expand in the presence of moisture after rinsing with phosphoric acid. However, with excessive air drying, the exposed collagen fibers tend to collapse, preventing the penetration of the adhesive due to the closed interfibrillar space, subsequently affecting the bonding success.^24^ Additionally, over-drying of the dentin surface causes an outward flow of dentinal fluid, which triggers nerve fibers around the odontoblastic process and prompts sensitivity.^37^ On the other hand, inadequate removal of the excess water on dentin after phosphoric acid rinsing can dilute the adhesive and compromise its structural integrity, penetration, and polymerization process.^13^

Universal single-step adhesive systems, on the other hand, were developed to address the limitations of the earlier adhesives and function effectively on different bonding substrates.^1,17,48^ These adhesive systems combine all the adhesive system components in one bottle as single-step adhesives, yet can be used in multi-mode etching protocols. In addition, some may contain a silane coupling agent and function as a glass-ceramic primer, which enables bonding to silica-based ceramic restorations.^33^ Moreover, manufacturers claim that the universal adhesives are chemically stable and not sensitive to different dentin moisture conditions, unlike one-step self-etch and the three-step etch-and-rinse bonding techniques.^1,48^ It was reported that the bond strength of Scotchbond Universal adhesive when applied in etch-and-rinse^20^ and in self-etch mode^16^ was not affected by the dentin surface moisture condition (wet or dry). Producing consistent bond strength regardless of the dentin condition is an essential characteristic of the selected adhesive system, as it indicates more predictable and less variable clinical performance.

One of the significant reasons for the advancement of adhesive systems is to simplify the application process and eliminate operator error. This has led to investigations into whether the operators’ variability and their level of clinical expertise correlate with the usage of single-step or multi-step adhesive systems. Earlier studies have revealed that there was variation in operators’ performance when applying etch-and-rinse compared to self-etch adhesives, which is attributed mainly to how the operators apply the adhesives in addition to understanding the definitions of different dentin moisture conditions (wet, moist and dry).^7,38,39,44^ Solderholm et al evaluated the influence of operators’ expertise on the bond strength of etch-and-rinse adhesive and self-etch adhesive systems and found that the experienced operators outperformed the inexperienced operators.^38^ Unlu et al reported similar results of higher shear bond strength values that were associated with an increased level of operators’ experience.^44^ Previous studies evaluated the performance of etch-and-rinse and self-etch adhesives when applied by various operators or under different dentin moisture conditions. However, no studies in the current literature reported the intercorrelation between operators’ variability in clinical experience, demonstration of the adhesive application technique as per the manufacturer’s instructions, dentin surface moisture condition, and the consistency of shear bond strength of universal adhesives. Therefore, the aim of this study was to determine the influence of operator experience, dentin moisture condition, and adhesive type on shear bond strength, and subsequently evaluate whether the adhesives performed reliably under these conditions.

## METHODS AND MATERIALS

Three hundred non-carious unidentified human posterior teeth were collected upon Institutional Review Board (IRB) approval, from the Periodontology and Oral Maxillo-Facial Surgery Departments at Nova Southeastern University, to obtain dentin substrates for experiments. The collected teeth were properly cleaned and stored in an aqueous disinfectant (Chloramine T, 0.5 wt% at 4°C).^21^

### Specimen Preparation

The collected teeth were sectioned using a slow-speed saw with a diamond disk under water cooling to separate the crowns from the roots at the cemento-enamel junction (CEJ) level. A buccolingual longitudinal section along the center of each crown was completed to expose dentin surfaces. The resulting two sections of each crown were randomly assigned to different experimental subgroups to ensure even distribution of specimens. Following sectioning, specimens were embedded in a chemically-polymerized methacrylate (Acratray, Autopolymerizable Custom Tray Material, Henry Schein, FL, USA) using the Ultradent Shear Bond Strength Test System’s mold (Ultradent, South Jordan, UT, USA) with the dentin surface exposed to fabricate cylindrical specimens. Specimens were then ground flat to expose the uncontaminated dentin surface as well as to create a parallel surface to the direction of the load cell using the Ultradent grinding plate and grinding mandrel (Ultradent, South Jordan, UT, USA) with water cooling. The specimen surfaces were subsequently finished with 400 and 600-grit SiC paper (Metaserv 2000 Variable Speed Grinder-Polisher, Lake Bluff, IL, USA) at a speed of 500 n/min. Then, all specimens were ultrasonically cleaned for 10 min before examination under the optical microscope (SZX7, Olympus, Tokyo, Japan) to ensure the absence of caries, demineralization, sclerotic dentin, cracks, and other pathologies. Specimens were stored in light-proof containers at 100% humidity in a 37°C incubator for 24 h before testing.

### Study Design and Groups

Specimens (n = 600) were randomly assigned to study groups with a sample size of five in each subgroup. The subgroups were categorized based on four independent variables: the type of adhesive system (2 types), the operator’s experience (2 experience levels), whether the application was performed before or after a demonstration of the technique (2 demonstration levels), and the moisture condition of the dentin surface (3 surface moisture conditions) (Table 1). This design resulted in 24 experimental subgroups (2 × 2 × 2 × 3). Each subgroup consisted of five dentin specimens (n = 5) bonded by a single operator. A total of 10 operators with varying levels of experience participated in the study: five first-year predoctoral dental students with no prior dental experience, and five dentists with more than 10 years of clinical practice, currently working at Nova Southeastern University. This resulted in a total of 600 specimens (2 × 2 × 2 × 3 × 5 samples ×5 operators per experience level). The adhesive systems that were tested were the universal adhesive (Adhese Universal, Ivoclar, Amherst, NY) and an etch-and-rinse adhesive (OptiBond FL, Kerr, Orange, CA) (Table 2).

**Table 1:** Experimental groups and dentin specimen distribution according to adhesive system, operator experience, demonstration of the application technique, and dentin moisture condition

Specimens (total n = 600)	Operator	Adhesive system	Demonstration	Dentin condition
*n* = 300 dentin specimens in Dentist group	**Dentists (5 dentists)** (*n* = 60/dentist)	**Etch and rinse adhesive** (*n* = 30)	Before demonstration (*n* = 15)	Wet (*n* = 5)
				Moist (*n* = 5)
				Dry (*n* = 5)
			After demonstration (*n* = 15)	Wet (*n* = 5)
				Moist (*n* = 5)
				Dry (*n* = 5)
		**Universal adhesive** (*n* = 30)	Before demonstration (*n* = 15)	Wet (*n* = 5)
				Moist (*n* = 5)
				Dry (*n* = 5)
			After demonstration (*n* = 15)	Wet (*n* = 5)
				Moist (*n* = 5)
				Dry (*n* = 5)
*n* =300 dentin specimens in Student group	**Students (5 students)** (*n* = 60/student)	**Etch and rinse adhesive** (*n* = 30)	Before demonstration (*n* = 15)	Wet (*n* = 5)
				Moist (*n* = 5)
				Dry (*n* = 5)
			After demonstration (*n* = 15)	Wet (*n* = 5)
				Moist (*n* = 5)
				Dry (*n* = 5)
		**Universal adhesive** (*n* = 30)	Before demonstration (*n* = 15)	Wet (*n* = 5)
				Moist (*n* = 5)
				Dry (*n* = 5)
			After demonstration (*n* = 15)	Wet (*n* = 5)
				Moist (*n* = 5)
				Dry (*n* = 5)


**Table 2:** A list of materials used in this study

Materials	Batch number	Composition	Manufacturer
Optibond FL Adhesive	Primer: 6759080 Adhesive: 6806169	Acid: 37% phosphoric acid Primer: ethanol, CQ, HEMA, GPDM, PAMM, water Adhesive: BIS-GMA, TEG-DMA, UDMA, GPDM, CQ, HEMA, filler	Kerr, Orange, CA, USA
Adhese Universal Adhesive	X28085	MDP, BIS-GMA, HEMA, MCAP D3MA, ethanol, water, initiator, silicon dioxide, stabilizers	Ivoclar, Amherst, NY, USA
Tetric EvoCeram Bulk Fill Composite resin	X40333	Resin matrix: BIS-GMA, BIS-EMA, UDMA Fillers: Ba–Al–Si-glass, pre-polymer filler (monomer, glass filler and ytterbium fluoride), spherical mixed oxide	Ivoclar, Amherst, NY, USA


**Fig 1:**
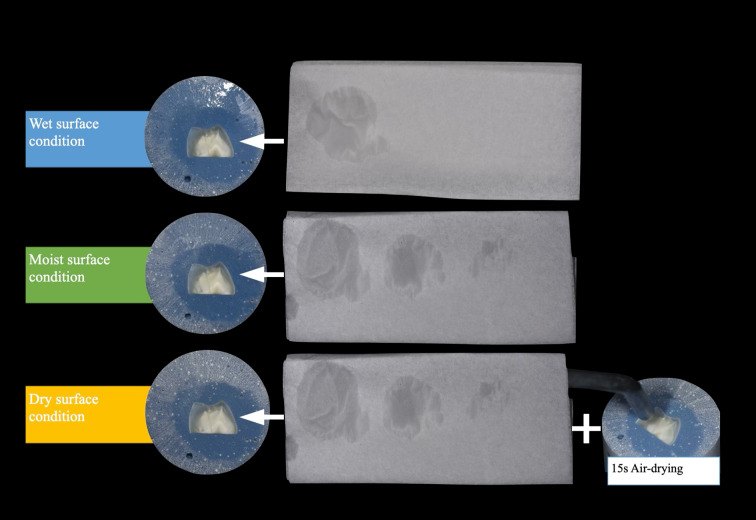
Dentin surface condition preparation process to obtain standard wet, moist, and dry conditions prior to adhesive application.

The study also included a demonstration effect comparison, where the operators applied the adhesives before and after receiving a demonstration of the manufacturer’s recommended adhesive application technique, provided by the principal investigator. The dentin surface condition was also controlled by the principal investigator and categorized into three levels based on the drying process: (1) wet, obtained after rinsing the samples with water and then dabbing once with the Kimwipe^TM^; (2) moist, obtained after rinsing with water and dabbing three times with the Kimwipe^TM^; and (3) dry, obtained after rinsing with water, dabbing three times with the Kimwipe^TM^, and then air drying for 15 s with an air syringe (Fig 1).

### Bonding Procedures

Before bonding, all operators (students and dentists) received brief instructions on the basic application steps for each adhesive system. The instruction included the number and sequence of steps for applying each adhesive system, as well as curing parameters and timing. No demonstration was provided at this stage. Afterward, operators applied the adhesives on specimens with three dentin moisture conditions (wet, moist, and dry). Following this, the principal investigator conducted a thorough live demonstration of the manufacturer’s recommended application technique. The demonstration covered the application methods, timing of each adhesive step, and the light-curing protocol. The same examiner, who was also the principal investigator, observed, timed, and took notes on the operators’ adhesive application process. Operators’ performance was also recorded on video for reference, with their prior consent obtained.

After adhesive application and polymerization, the specimens were stored in sealed dark containers at room temperature to prevent exposure to light. A small amount of distilled water was placed at the bottom of these containers to ensure 100% humidity, with specimens positioned above the water level to avoid direct contact with water and disturbance of the hybrid layer. Composite was applied by the principal investigator within 10 min of adhesive curing, ensuring standardized application among all groups.

### Composite Resin Application

**Fig 2a and b:**
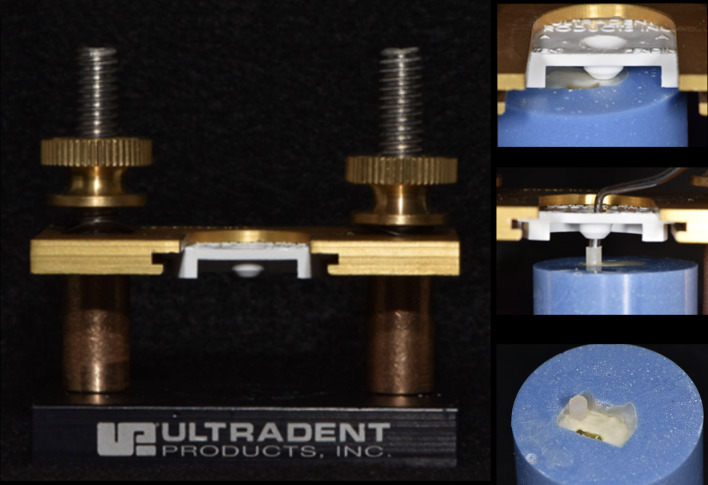

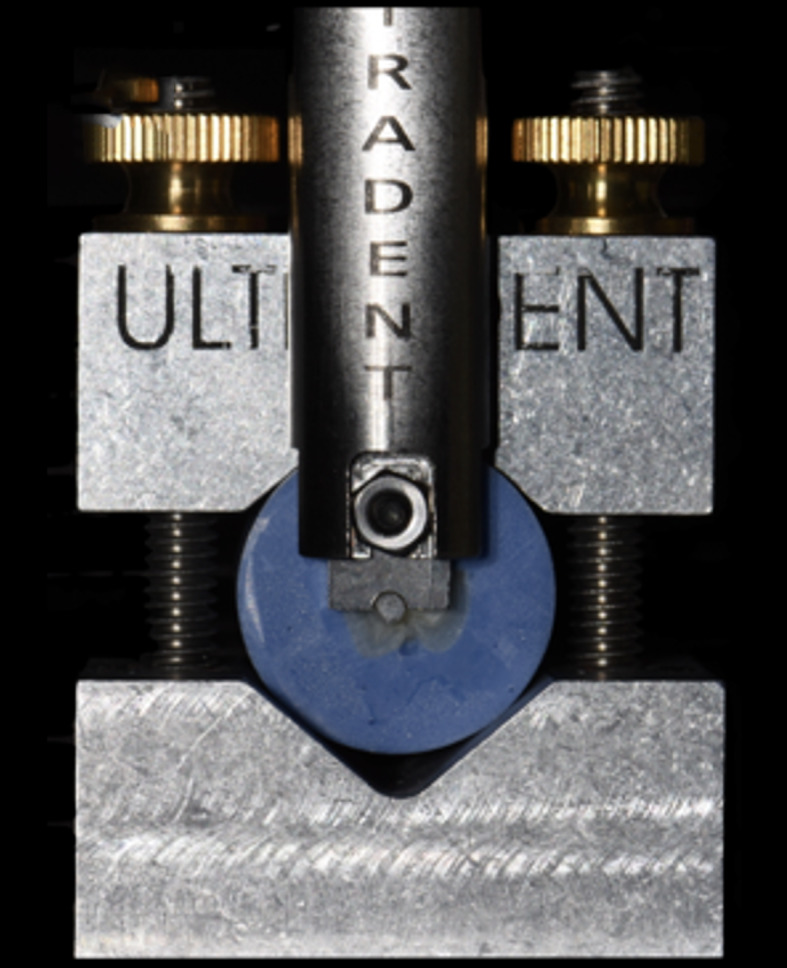
Armamentarium used for shear bond strength test. (a) Ultradent Shear Bond Jig and (b) Testing application assembly.

Specimens were placed on a custom-fabricated shear bond jig in 2.38 mm diameter (Ultradent, South Jordan, UT, USA) by the principal investigator. The Teflon mold of the bonding jig was filled with composite resin (Tetric EvoCeram Bulk Fill, Shade IVA, Ivoclar, Amherst, NY, USA) in a single increment (t =2 mm, d =2 mm) (Fig 2a). A light-emitting diode (LED) curing light with the intensity of 1,200 mW/cm^2^ and a wavelength of 385–515 nm (Bluephase G2, Ivoclar, Amherst, NY, USA) was used to polymerize the composite resins for 20 s. The light-cure intensity was monitored between specimens (with an interval of 10 specimens) to maintain the intensity of 1,200 mW/cm^2^ using a calibrated radiometer (Bluephase Meter II, Ivoclar, Amherst, NY, USA). Specimens were stored in the incubator 100% humidity for 24 h at 37°C immediately after composite bonding, with specimens positioned above water level to avoid direct contact with water.

### Shear Bond Strength (SBS) Testing

A universal testing machine (Instron, Canton, MA, USA) was used to determine the shear bond strength at a cross-head speed of 1 mm/min with a load cell of 1,000 lbs. Specimens were stabilized in an Ultradent testing jig and then positioned so that the notched cross-head load cell was placed precisely against the specimen’s bonded surface, applying the load parallel to the long axis of the specimen (Fig 2b). The load required to debond the specimen was recorded and expressed in megapascals (MPa) by dividing the load by the surface area of the bonded specimen. The mean SBS values per study group were calculated.^32^

### Failure Analysis

Optical and scanning electron microscopes (SEM) were used to analyze the interface between the dentin surface and composite after the SBS test to determine the pattern of bond failure. The energy dispersive spectroscopy (EDS) technology in SEM was utilized to identify the specific elements to confirm the failure mode. Failures were classified either as an adhesive failure between the dentin surface/the adhesive layer, or cohesive failure within the composite resin, adhesive or dentin, or mixed failures involving multiple structures.

### Statistical Analysis

A Log Worth test was used to identify the factors that affect the bond strength of the composite resin to the dentin surface. Tukey HSD adjustment was used for pairwise comparisons to identify significant differences in bond strength by adhesives, operators, and dentin moisture condition. A boxplot was used to display the median, interquartile range (IQR), and distribution of shear bond strength values, including outliers, for both etch-and-rinse and universal adhesives.

## RESULTS

### Shear Bond Strength Test

The Log Worth test revealed that the adhesive type (universal and etch-and-rinse adhesives) was the most critical factor in determining bond strength of composite resin restorations to dentin, followed by surface moisture condition (wet, moist, and dry), and operators’ experience variability (students and dentists) (*P* < 0.05), while demonstration of the adhesive application technique had no significant effect. Furthermore, the interaction between the adhesives and dentin moisture condition had a significant effect on the bond strength (*P* < 0.05). The combined effect of adhesive type, demonstration, and operator experience also significantly influenced the bond strength (*P* < 000.1) (Table 3).

**Table 3:** Log Worth test

Source	Log Worth	*P* value
Adhesive	19.96	0.000
Adhesive*Demonstration*Operator	4.24	0.000
Condition	3.73	0.000
Adhesive*Condition	3.58	0.000
Operator	1.52	0.030
Adhesive* Operator	1.09	0.082
Adhesive* Operator *Condition	0.64	0.228
Demonstration* Operator *Condition	0.47	0.339
Adhesive*Demonstration	0.33	0.467
Operator *Condition	0.19	0.654
Demonstration* Operator	0.15	0.716
Demonstration*Condition	0.11	0.770
Adhesive*Demonstration* Operator *Condition	0.09	0.805
Demonstration	0.09	0.812
Adhesive*Demonstration*Condition	0.02	0.949


Tables 4a and 4b illustrate the model effects and statistical pairwise comparisons using a Tukey HSD adjustment. A significant difference in bond strength by adhesives, operators, and dentin moisture condition was found. The universal adhesive achieved a dentin bond strength 7.38 ± 0.73 MPa higher than that of the etch-and-rinse adhesive (*P* < 0.0001), regardless of operators’ expertise, dentin moisture conditions, or demonstration of the adhesive application technique. Moreover, dentists obtained 1.65 ± 0.73 MPa higher dentin bond strength than students. Regarding the dentin moisture condition, dry and moist surfaces resulted in significantly higher bond strength by 3.79 ± 0.89 (*P* < 0.0001) and 2.67 ± 0.89 MPa (*P* < 0.002), respectively, compared to wet surfaces. However, there was no significant difference between dry and moist surfaces (*P* = 0.213). The descriptive statistics of shear bond strength by the combination of factors of adhesive type, operator experience, technique demonstration, and dentin moisture condition are presented in Table 5.

**Table 4a:** Model effects

Source	DF	Sum of squares	F ratio	Prob > F
Adhesive	1	8169.14	93.99	<.0001
Demonstration	1	4.92	0.06	0.812
Adhesive*Demonstration	1	46.02	0.53	0.467
Operator	1	410.34	4.72	0.030
Adhesive* Operator	1	263.78	3.04	0.082
Demonstration* Operator	1	11.50	0.13	0.716
Adhesive*Demonstration* Operator	1	1428.85	16.44	< 0.0001
Condition	2	1517.11	8.73	< 0.0001
Adhesive*Condition	2	1452.43	8.36	< 0.0001
Demonstration*Condition	2	45.51	0.26	0.770
Adhesive*Demonstration*Condition	2	9.19	0.05	0.949
Operator *Condition	2	73.98	0.43	0.654
Adhesive* Operator *Condition	2	257.42	1.48	0.228
Demonstration* Operator *Condition	2	188.38	1.08	0.339
Adhesive*Demonstration*Operator*Condition	2	37.62	0.22	0.806


**Table 4b:** Pairwise comparisons by main effects

			Difference	SE	95% Lower CL	95% Upper CL	*P* value
Dentist	Vs.	Student	1.65	0.78	0.13	3.18	0.033
After demonstration	Vs.	Before demonstration	0.18	0.73	–1.25	1.62	0.804
Universal adhesive	Vs.	Etch & rinse adhesive	7.38	0.73	5.94	8.81	< 0.0001
Dry	Vs.	Wet	3.79	0.89	2.03	5.55	< 0.0001
Moist	Vs.	Wet	2.67	0.89	0.92	4.43	0.002
Dry	Vs.	Moist	1.12	0.89	-0.64	2.87	0.213


**Table 5:** Descriptive statistics of shear bond strength by adhesive type, operator experience, technique demonstration, and dentin moisture condition

Adhesive	Demonstration	Operator	Condition	Mean	SE	Lower 95% CI	Upper 95% CI
Etch & rinse adhesive	After	Dentist	Dry	23.77	1.86	20.10	27.43
Etch & rinse adhesive	After	Dentist	Moist	22.21	1.86	18.55	25.87
Etch & rinse adhesive	After	Dentist	Wet	16.38	1.86	12.72	20.05
Etch & rinse adhesive	After	Student	Dry	18.05	1.86	14.39	21.71
Etch & rinse adhesive	After	Student	Moist	14.70	1.86	11.04	18.36
Etch & rinse adhesive	After	Student	Wet	10.58	1.86	6.92	14.24
Etch & rinse adhesive	Before	Dentist	Dry	19.71	1.86	16.05	23.37
Etch & rinse adhesive	Before	Dentist	Moist	19.17	1.86	15.51	22.83
Etch & rinse adhesive	Before	Dentist	Wet	14.50	1.86	10.84	18.17
Etch & rinse adhesive	Before	Student	Dry	24.14	1.86	20.48	27.80
Etch & rinse adhesive	Before	Student	Moist	16.56	1.86	12.89	20.22
Etch & rinse adhesive	Before	Student	Wet	13.84	1.86	10.18	17.50
Universal adhesive	After	Dentist	Dry	24.37	1.86	20.70	28.03
Universal adhesive	After	Dentist	Moist	23.59	1.86	19.92	27.25
Universal adhesive	After	Dentist	Wet	24.97	1.86	21.31	28.63
Universal adhesive	After	Student	Dry	25.34	1.86	21.67	29.00
Universal adhesive	After	Student	Moist	29.26	1.86	25.60	32.92
Universal adhesive	After	Student	Wet	25.77	1.86	22.11	29.43
Universal adhesive	Before	Dentist	Dry	26.02	1.86	22.36	29.68
Universal adhesive	Before	Dentist	Moist	27.01	1.86	23.35	30.67
Universal adhesive	Before	Dentist	Wet	26.11	1.86	22.45	29.77
Universal adhesive	Before	Student	Dry	23.61	1.86	19.95	27.28
Universal adhesive	Before	Student	Moist	23.59	1.86	19.93	27.25
Universal adhesive	Before	Student	Wet	22.53	1.86	18.87	26.19


**Fig 3a to c:**
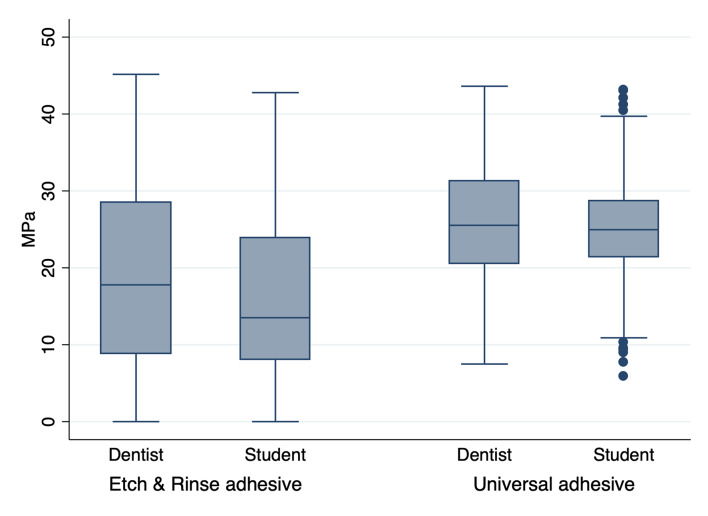

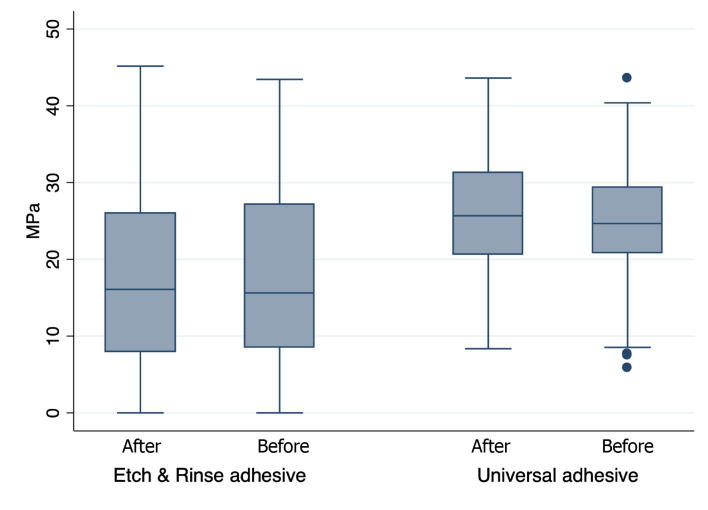

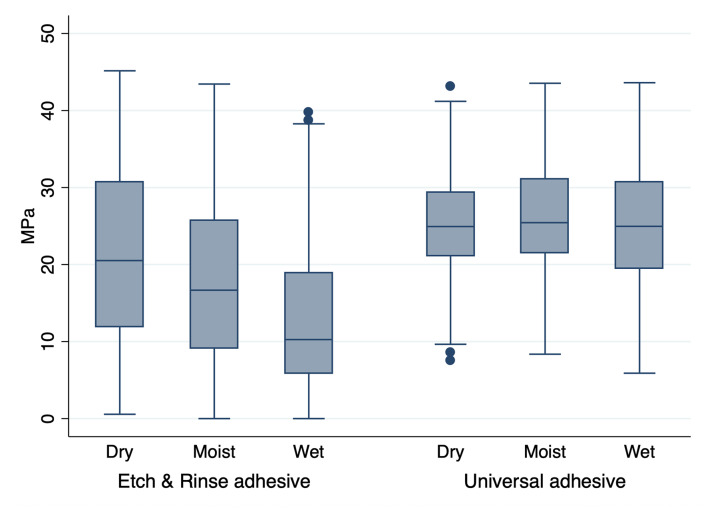
Box-plot graph of adhesives showing cumulative shear bond strength results by adhesive system and (a) operator experience, (b) demonstration of the adhesive application technique, as well as (c) dentin moisture condition.

Figure 3 illustrates a boxplot displaying the median, interquartile range (IQR), and distribution of shear bond strength values, including outliers, for both etch-and-rinse and universal adhesives. The data in Figure 3a showed that the median bond strength of etch-and-rinse adhesive obtained by dentists was 17.79 MPa with IQR 19.61 MPa, while students showed a lower median of 13.52 MPa with IQR 15.81 MPa. On the other hand, the universal adhesive exhibited a comparable median bond strength when applied by dentists versus students (25.53 MPa and 24.96 MPa, respectively), with narrower IQRs of 10.6 MPa and 7.3 MPa, respectively, indicating consistency in bond strength outcomes and reduced variability across operators. Complete data for all subgroups are presented in Figures 3b and 3c.

Overall, these findings indicate that the universal adhesive exhibits a higher and more consistent bond strength than the etch-and-rinse adhesive, with reduced sensitivity to operator variability and dentin moisture condition.

### Failure Analysis

**Fig 4a to e:**
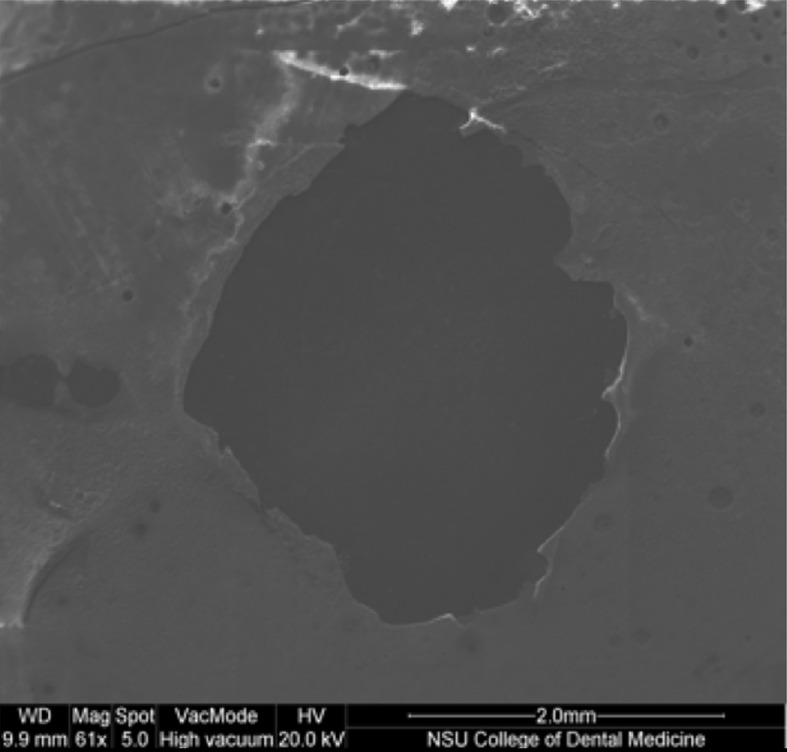

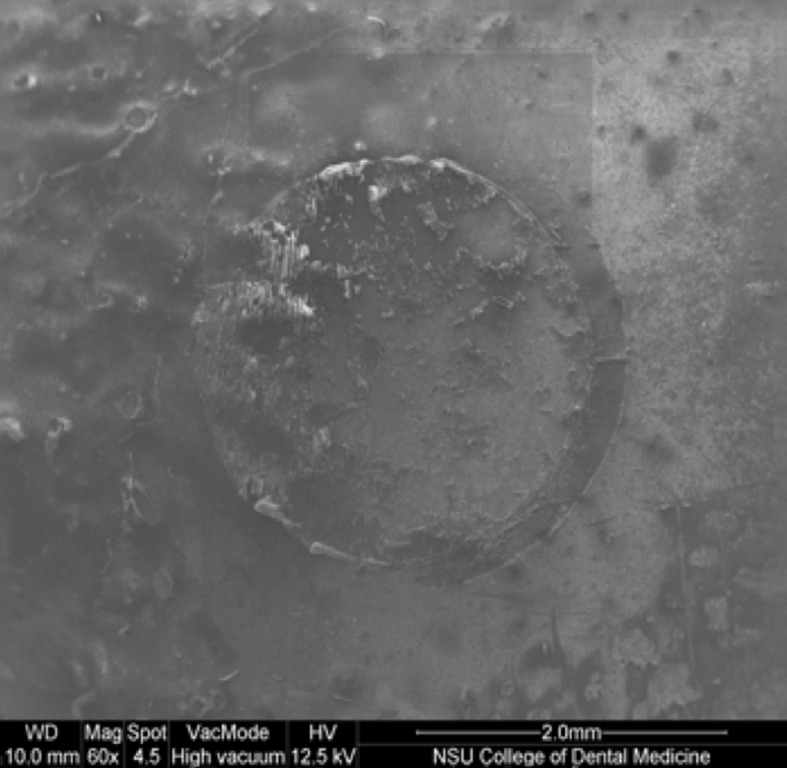

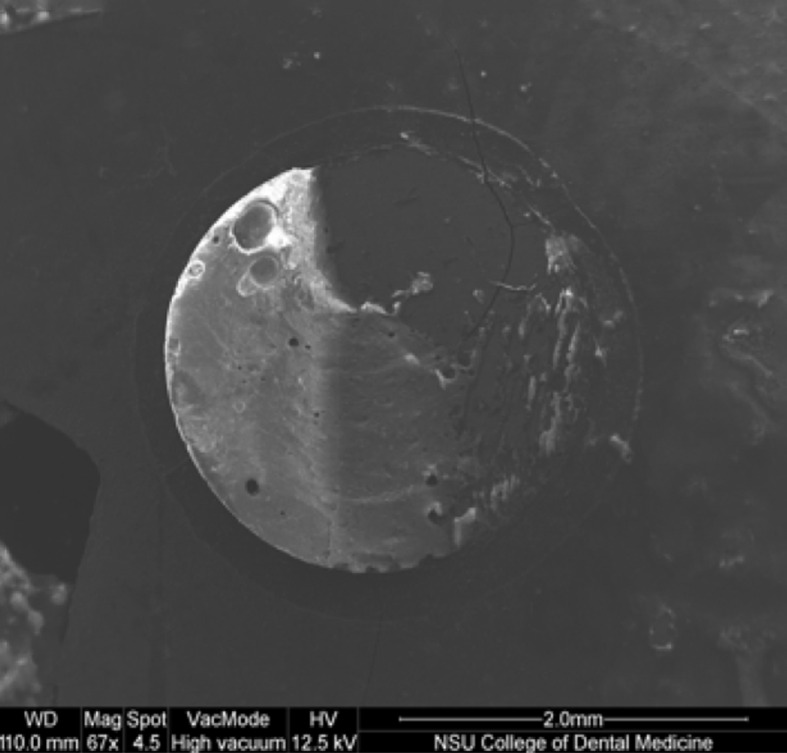

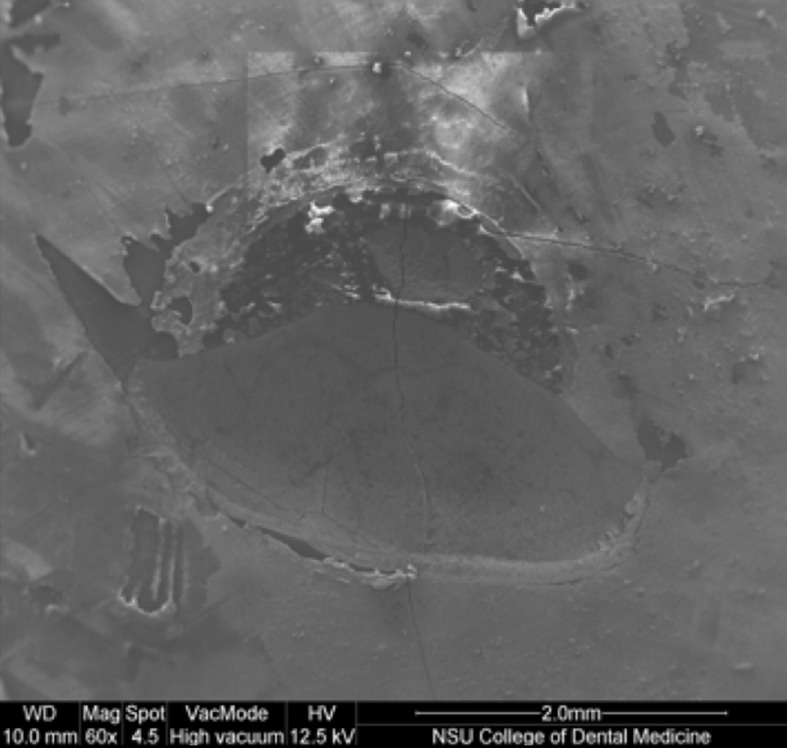

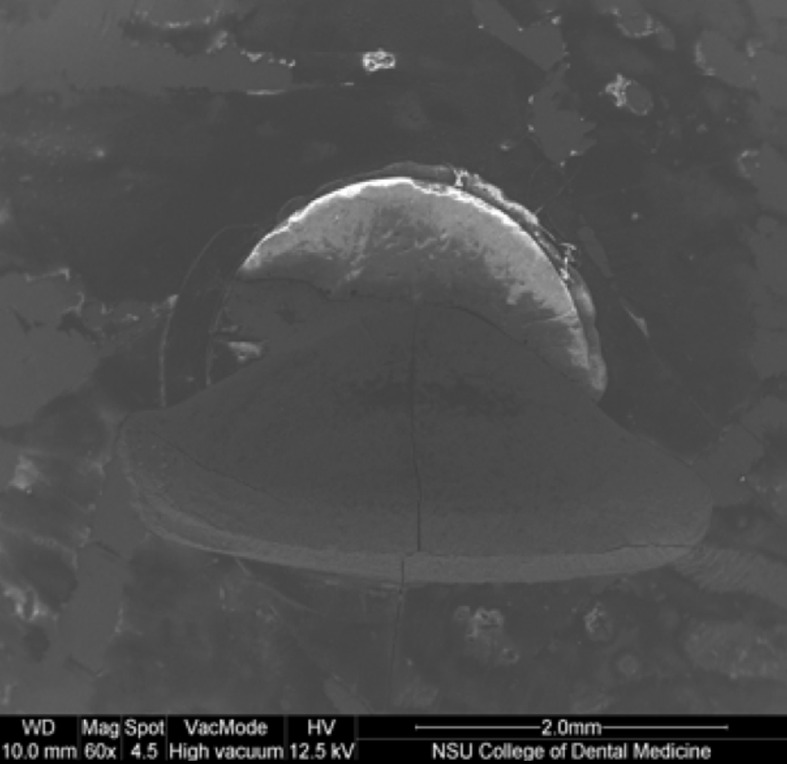
Failure mode identification and analysis using scanning electron microscope (SEM). Representative SEM images of (a) an adhesive failure, (b) a cohesive failure within the adhesive layer, (c) a cohesive failure in composite, (d) a cohesive failure in dentin, (e) a mixed failure.

After determining the failure modes associated with each adhesive system, distinct patterns emerged. In the etch-and-rinse group, adhesive failure was the most common (30%), and 51% of these adhesive failures were observed with wet dentin surfaces. The second common failure was cohesive in the composite (27%), followed by cohesive failure within the adhesive layer (24%). In contrast, the universal adhesive exhibited primarily cohesive failure in the composite (47%), followed by mixed failure (28%), with adhesive failure being the least common (6%). Detailed descriptive statistics of failure modes for each group are shown in Table 6. Additionally, representative SEM images of the resin-dentin interfaces corresponding to each failure mode are illustrated in Figure 4.

**Table 6:** Descriptive statistics of failure modes across adhesive type, operator experience, technique demonstration, and dentin moisture condition

			After demonstration		Before demonstration	
			Etch and rinse adhesive	Universal adhesive	Etch and rinse adhesive	Universal adhesive
**Dentist**	Dry	A	5	2	6	2
		CA	3	3	5	0
		CC	7	13	9	16
		Mix	8	6	5	6
		CD	2	1	0	1
	Moist	A	6	2	6	0
		CA	4	5	6	1
		CC	6	11	7	13
		Mix	9	6	4	11
		CD	0	1	2	0
	Wet	A	11	2	10	2
		CA	1	5	5	3
		CC	12	11	4	11
		Mix	1	6	4	8
		CD	0	1	2	1
**Student**	Dry	A	8	0	2	1
		CA	7	4	7	5
		CC	7	8	7	12
		Mix	3	8	8	5
		CD	0	2	1	4
	Moist	A	6	1	5	4
		CA	12	4	11	5
		CC	7	12	5	11
		Mix	0	8	3	4
		CD	0	0	1	1
	Wet	A	12	1	13	2
		CA	8	1	3	4
		CC	4	16	6	7
		Mix	0	6	3	12
		CD	1	1	0	0
A: indicates an adhesive failure, CA: cohesive failure within the adhesive layer, CC: cohesive failure in composite, Mix: mixed failure, CD: cohesive in dentin.						

**Fig 5a to d:**
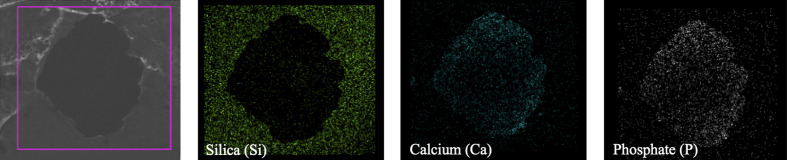

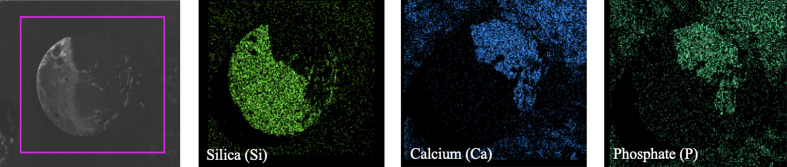

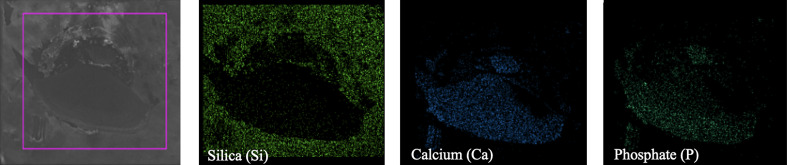

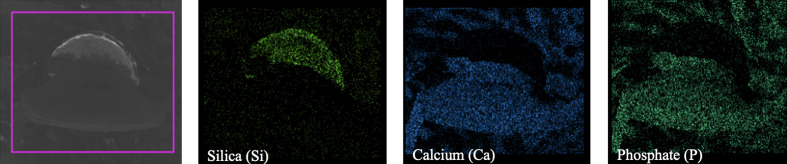
Energy dispersive spectroscopy (EDS) for failure mode identification. Elemental mapping of (a) an adhesive failure, (b) a cohesive failure in composite, (c) a cohesive failure in dentin, (d) a mixed failure.

EDS elemental mapping showed elements that represent the dentin surface and the adhesive and composite resin materials, providing further confirmation of the adhesive failure mode observed after shear bond strength tests. The EDS elemental mapping highlighted calcium (C) and phosphate (P) elements on the resin/dentin interface at the bonding site with no presence of silica (Si), aluminum (Al), or ytterbium (Yb), indicating an adhesive mode of failure (Fig 5a). The fully saturated areas of Si and Al at the bonding site were an indication of a cohesive failure in the composite resin, while cohesive failure in dentin was mainly associated with saturated areas of P and C (Figs 5b and 5c). On the other hand, the mixed mode of failure exhibited two distinct areas: one characterized by a high concentration of C and P, and the other by a high concentration of Si and Al. These areas signify a mixture of fractured tooth structure and fractured composite resin at the bonding interfaces (Fig 5d). Elemental mapping was unable to detect ytterbium in the used composite resin as it was below the detection level in all tested specimens.

## DISCUSSION

Although etch-and-rinse adhesives were considered the gold standard for bonding composite resins to dentin, multiple clinical steps and the sensitivity of their application technique present numerous opportunities for errors to occur.^45^ Thus, the present study evaluated the effect of operator experience on the shear bond strength of composite resins to dentin, comparing a multi-step etch-and-rinse adhesive with a single-step universal adhesive. In addition, this study investigated the influence of dentin surface moisture conditions and the demonstration of adhesive application techniques on their performance in terms of shear bond strength. The results of this study revealed that the universal adhesive (Adhese Universal) obtained significantly higher and more consistent dentin bond strengths (MPa) than the etch-and-rinse adhesive (Optibond FL), considering the variables of operator experience, dentin moisture condition, and application technique demonstration. In agreement with our findings, Patidar et al found that another universal adhesive (Scotchbond Universal, 3M ESPE) had a significantly higher bond strength than etch-and-rinse adhesive (Adper Single Bond 2, 3M ESPE).^27^ In other studies, universal adhesives (Scotchbond Universal and G-Premio Bond) achieved a comparable but not significantly different bond strength to dentin in self-etch mode as etch-and-rinse adhesives (Adper Single Bond 2).^10,36^ Two to three coats of etch-and-rinse adhesives were applied in some of these studies,^10,11^ which may explain the higher bond strength values than those reported in this study. Our results revealed significant interactions between the independent variables of operator, technique demonstration with adhesive type, as well as between dentin moisture condition and adhesive type (*P* < 0.05). This indicates that the lower dentin bond strength observed with the etch-and-rinse adhesive may be attributed to its sensitivity to variations in dentin moisture conditions and operator experience in application technique.^11,23^ Unlike the single-step universal adhesive, the etch-and-rinse adhesive application technique involves multiple clinical steps, including etching, rinsing, drying, priming, and adhesive placement, which has been shown to introduce the potential for technique variability and increase the chances of error.^44^

This study shows that not only the adhesive type but also the dentin moisture control significantly affected dentin bond strength. In particular, wet surfaces consistently yielded lower values compared to dry and moist dentin surfaces, with the difference being significantly greater with the etch-and-rinse adhesive. However, the bond strength values remained unaffected by dentin dryness compared to moist surface conditions with both water-containing adhesive systems tested. The water content of these adhesives was found to enable the re-expansion of collapsed fibers, thereby facilitating the penetration of the adhesive and increasing bond strength to dentin.^8,12^ Van Meerbeek confirmed that the dry-bonded water-based adhesives produce a hybrid layer without ultrastructural evidence of collapsed dentin collagen fibers.^46^ Other studies also agreed with this study, further confirming that water-based primers provide comparable bond strength values on dry dentin as on moist dentin, provided that the primer is scrubbed afterward to evaporate the residual water, and no remaining water is entrapped within the hybrid layer.^6,14^

However, unlike the universal adhesives, the etch-and-rinse adhesive exhibited sensitivity to wet dentin. Our results clearly showed that lower bond strengths and a higher incidence of adhesive failures were associated with the application of etch-and-rinse adhesive to wet dentin surfaces compared to other dentin moisture conditions. For water/solvent and water-based adhesives, applying additional amounts of water to the dentin surface before the adhesive system was shown to significantly decrease bond strength,^30^ mainly due to dilution and compromised polymerization of the adhesive.^13^ However, although the tested universal adhesive was a water-based adhesive, it demonstrated insensitivity to variations in dentin moisture conditions (moist vs dry vs wet) and consistently yielded high shear bond strengths. This insensitivity likely relates to the advanced chemical formulation of universal adhesives^5^ and less variability in their application technique compared to etch-and-rinse adhesives.^9^ The universal adhesive, used in this study, contained ethanol and water solvents, which play a role in overcoming the dentin moisture difference. Water helps to re-expand the collapsed collagen fibers and permit the diffusion of monomer into the collagen network in case of an over-dried dentin surface,^26^ while ethanol promotes the penetration of the adhesive into exposed collagen fibers by facilitating the evaporation of the excess water in case of a wet dentin surface.^4^ Previous studies have found that in self-etch mode, alcohol/water-based universal adhesives do not exhibit a significant difference in bond strength under different moisture conditions compared to universal adhesives that do not contain water in their composition.^5,41,42^ All of this information leads to the conclusion that the insensitivity to dentin surface moisture is conditional and reliant on the adhesive formulation.

The influence of operator variability on shear bond strength of universal and etch-and-rinse adhesives was a major focus of this study. Specifically, whether differences in clinical experience among operators resulted in variations in the bonding application technique, ultimately affecting the bond strength outcomes of the adhesives investigated. Results showed that dentists with more than 10 years of clinical experience achieved higher dentin bond strength values than inexperienced students when they applied the etch-and-rinse adhesive. However, such a difference in bond strength was not detected within the universal adhesive group. Other studies reported that the difference in outcomes between experienced and inexperienced operators was primarily attributed to the experienced operator’s understanding of the concepts underlying the manufacturer’s bonding instructions for scrubbing, air drying, and re-wetting the surface with the adhesive.^38,39,44^ Our observations of the participating operators during their adhesive application process revealed that dentists tend to scrub the etch-and-rinse adhesive against the dentin surface meticulously and vigorously compared to students. Moreover, most dentists applied a continuous and strong air stream on the primer. Others waited for a couple of seconds between the primer and adhesive application steps. Previous research has confirmed that vigorous scrubbing and the air drying of the etch-and-rinse adhesive in the priming step increased the bond strength as they promote the solvent evaporation and prevent its entrapment in the hybrid layer.^6,14^

Interestingly, the universal adhesive showed no difference in bond strength between dentists and students, which could be attributed to the simplified application protocol and reduced number of application steps. The observations and records of the investigator in this study indicated that all operators scrubbed the universal adhesive for around 20 s, which was the time recommended by the manufacturer, even before the demonstration of the application technique. Earlier findings by Latta et al support that actively applying and scrubbing the adhesive against the dentin surface increases the bond strength of universal adhesives.^22^ As this process helps the adhesive penetrate the smear layer and induces solvent evaporation, it also facilitates the formation of more nano-layered calcium-salts at the interface with dentin.^22^ In agreement, Shafiei et al reported insusceptibility of universal adhesives to operator variability and found that microleakage at the restoration’s margin with universal adhesives in self-etch mode was not significantly different between dentists and students.^35^ However, in the Ueda et al study, it was observed that operators with clinical experience achieved lower dentin bond strength using self-etch composite cement but with higher consistency in the outcomes compared to inexperienced dental students.^43^ Ueda et al relate this finding to the application technique and careful adherence to application protocols by inexperienced dental students, which can be more influential than extensive clinical experience in achieving adequate bond strength to dentin.^43^ This study investigated whether demonstrating the application technique, through clear step-by-step instructions and live demonstration, could enhance operators’ adhesive application skills and result in a stronger bond. Although subgroups of students and dentists received a standardized demonstration of application protocols for etch-and-rinse and universal adhesives, this demonstration did not lead to a statistically significant improvement in dentin bond strength with either adhesive system. Notably, dentists performed better than students regardless of the demonstration when using the etch-and-rinse adhesive system. This implies that correct adhesive application technique, such as adhesive scrubbing and air drying, is not only a result of learning and observing the technique but rather develops over years of clinical experience and repeated practice.^2^

In summary, this study aimed to simulate multiple clinical scenarios and conditions to evaluate the consistency of performance of universal versus etch-and-rinse adhesives. The results of this study validated that the universal adhesive could achieve not only high bond strength but also consistent outcomes, regardless of variability in dentin moisture conditions and differences in clinical experience among operators. These findings enhance our understanding of how to achieve a more predictable and efficient bonding process that is less susceptible to operator error and variation in bonded substrate moisture conditions, leading to reliability as a function of these clinical variables. Considering that the tested universal adhesive has a unique formula compared to other products in the same category, the results of this study cannot be generalized. Future studies should investigate the performance of various universal adhesives under standard testing conditions and compare their results. Also, enhancing our understanding of their bonding behavior after aging is strongly indicated, as it reflects the reliability of these adhesives in the long term.

## CONCLUSION

Results of this study revealed significant interactions between the adhesive systems, operators’ expertise, and dentin moisture conditions. The tested universal adhesive demonstrated superior and consistent performance compared to the three-step etch-and-rinse adhesive, particularly given the variability among operators and dentin moisture conditions. It proved to be insusceptible to differences in the operator’s expertise or dentin moisture condition, whereas the etch-and-rinse adhesive was influenced by such variations. In conclusion, the tested universal adhesive demonstrated a reliable bond strength in comparison to the three-step etch-and-rinse adhesive. Additional studies are needed to show performance variation between the universal adhesives, knowing there are significant variations in universal adhesives’ compositions.

### Acknowledgments

The authors gratefully acknowledge the participants in this research project. NSU HPD research grant (#335920) and Ivoclar provided funding for this project. This study was part of a Master’s of Science thesis project of the first author.

#### Clinical relevance

The ability of the tested universal adhesive to achieve a strong bond consistently to dentin, regardless of the different operators’ expertise and bonded surface moisture conditions, makes the bonding process more predictable and less susceptible to error, thus reliable.
